# Implicit theories shape responses to social-evaluative threat

**DOI:** 10.3389/fpsyg.2023.1105721

**Published:** 2023-04-27

**Authors:** Máire B. Ford

**Affiliations:** Department of Psychological Science, Loyola Marymount University, Los Angeles, CA, United States

**Keywords:** social-evaluative threat, social threat, implicit theories, mindset, rumination, self-esteem

## Abstract

It is important to understand factors that make one more or less vulnerable to the harmful effects of social threat. This study focuses on the role of implicit theories (also referred to as mindsets) in shaping responses to a potent form of social threat, namely social-evaluative threat (SET). 124 individuals participated in an experimental study in which they were induced to have an incremental theory or an entity theory about their social skills. Next, they were exposed to SET in the laboratory. Psychological and physiological responses were assessed including social self-esteem, rumination, spontaneous mentions of concerns about one’s social skills, and heart-rate variability. Compared to those induced to have entity theories, those induced to have incremental theories were buffered from the typical harmful effects of SET on social self-esteem, rumination, and concerns about their social skills. The association between implicit theories and heart-rate variability fell just short of significance.

## Introduction

Social-evaluative threat (SET) involves a threat to one’s social or relational value. It is a common and potent social stressor and is associated with negative psychological and physical outcomes (Dickerson and Kemeny, 2004; Dickerson et al., 2008). In the current study, implicit theories were examined as a possible moderator of the effects of SET on psychological and physiological responses. Implicit theories are general beliefs about the malleability of personal traits (Burnette, et al., 2013). A large body of literature on implicit theories (for a review see Dweck, 2012) suggests that those who subscribe to incremental theories (those who believe that personal traits are malleable and can be improved upon) experience more positive outcomes in the face of challenging situations when compared to those who subscribe to entity theories (those who believe that they are born with certain fixed traits that are immutable to change). However, few researchers have utilized an experimental methodology to investigate the impact of implicit theories on responses to *social* or *relationship* events (for exceptions see Howe and Dweck, 2016; Maxwell et al., 2017). Because implicit theories tend to be domain-specific it is important to thoroughly investigate them in the context of social situations in order to determine whether and how they may impact outcomes in social domains. The current study seeks to contribute to research in this area by investigating the role that entity and incremental theories about one’s social skills play in shaping one’s responses to a common and meaningful type of social threat, namely SET.

Social relationships help us meet evolved needs for acceptance and belonging (Baumeister and Leary, 1995) and they provide us with numerous benefits, such as social support, and access to resources (Leary and Acosta, 2018). As such, events that threaten social relationships or suggest possible social devaluation are experienced as aversive and have harmful effects on one’s cognitions, emotions, behaviors, and physiology (Leary et al., 1995; Stroud et al., 2000; Dickerson and Kemeny, 2004; Zadro et al., 2004; Blackhart et al., 2007; Martin et al., 2018). SET is one such event. SET involves a threat to social/relational value, and it occurs in situations where an important aspect of oneself is or could be negatively judged by others (Dickerson et al., 2008). Examples of SET would include giving a speech in front of a harsh audience, or interacting with an individual who appears to be judging us negatively. In studies comparing SET to other stressors, SETs have been found to have especially detrimental effects (Dickerson and Kemeny, 2004; Dickerson et al., 2008). Some of the negative outcomes associated with SET include increases in state rumination (Zoccola et al., 2008), negative emotions (Lehman et al., 2015), cardiovascular reactivity (Christian and Stoney, 2006), systolic blood pressure reactivity (Smith et al., 1997; Lehman et al., 2015), pro-inflammatory cytokine activity (Dickerson et al., 2009), increased cortisol reactivity (Dickerson and Kemeny, 2004; Dickerson et al., 2008; Bosch et al., 2009), and decreased social self-esteem (Gruenewald et al., 2004). Over time, detrimental responses to SET may accumulate, and may contribute to decrements in physical and psychological health.

Although SET is normatively distressing, some individuals are more resilient in the face of SET. One factor that most certainly plays an important role in one’s response to SET is one’s appraisal of the event. According to the appraisal theory of emotion (e.g., Lazarus et al., 1985) and the biopsychosocial model of challenge and threat (for a review see Blascovich and Mendes, 2010) one’s appraisal of a given stressor can have important implications for psychological and physiological responses to the stressor. Thus, it is important to consider factors that might affect appraisals of SET, such as implicit theories (also sometimes referred to as mindset), which have been found to shape appraisals of demanding or stressful situations (Burnette, et al., 2013).

As mentioned above, an individual’s implicit theories tend to fall into one of two categories: (1) entity theorists believe that a given trait is fixed and cannot change, and (2) incremental theorists believe that a given trait can be changed with practice and effort. Entity theorists believe that they do not have the skills necessary to succeed in specific challenging situations and that it is not possible for them to develop these skills. They exhibit less resilience and tend to pursue more defensive strategies (Nussbaum and Dweck, 2008). In contrast, incremental theorists interpret adversity as an opportunity for growth. They believe that with effort they can develop the necessary skills to succeed and when faced with a setback they tend to display persistence. It is reasonable to believe that implicit theories might play an important role in shaping responses to SETs. First, because implicit theories have the greatest effect on outcomes in the context of a challenging event (Dweck, 2012), this makes them prime candidates for investigation in the context of SET. Additionally, in situations where there is an ego threat (a threat to one’s positive self-image or sense of self-worth) implicit theories seem to play an especially important role in shaping responses. For example, results from a meta-analysis suggest a moderating effect of ego threat on the relationship between implicit beliefs and self-regulation (Burnette et al., 2013) whereby implicit theories were more likely to predict one’s responses when ego threat was present versus when it was not present.

Although the impact of implicit theories on outcomes in achievement/problem-solving situations has been well-investigated less work has been done to investigate how implicit theories might operate in social situations. Additionally, most of the work done investigating the link between implicit theories and social situations has investigated these processes in adolescents (Yeager and Miu, 2011; Yeager et al., 2014, 2016). Findings from the small number of studies conducted on adults suggest that incremental theories may buffer individuals from the negative effects of social adversities. For example, researchers found that adults’ incremental theories about relationships (believing that successful relationships are developed over time versus believing in relationship destiny) were associated with healthy coping strategies in response to negative relationship events (Knee, 1998). Howe and Dweck (2016) found that adults with entity theories of personality responded to imagined or recalled rejection with lingering negative affect, negative self-definition, and fear of recurrence of rejection. Thus, implicit theories seem to shape responses to social situations and they may play an important role in responses to SET.

The current investigation will fill certain gaps in the literature on the role of implicit theories in shaping responses to SET. Current studies have largely focused on one’s pre-existing incremental/entity theories and/or have primarily relied on imagined or recalled SETs rather than looking at responses to actual SETs in real time. Experiments that have manipulated implicit theories and then investigated actual responses to SETs have primarily been conducted on children and adolescents and have focused on incremental theories and not entity theories. Thus, these studies typically manipulate only incremental theories and not entity theories (Yeager et al., 2014). The current study seeks to fill this gap in the literature by investigating the role of *both* incremental and entity theories in shaping responses to a real-time SET created in the laboratory. Thus, the current study will allow for causal claims about the effects of implicit theories on responses to an actual SET and both incremental and entity theories will be investigated. Additionally, given that implicit theories are domain-specific (e.g., a person may have an entity theory about intelligence but an incremental theory about athletics), the current study will hone in on specific implicit theories that are most relevant in a situation of SET, namely implicit theories about social skills. These implicit theories should be more proximal and robust predictors of responses to SET than broader implicit theories (such as implicit theories about intelligence or personality).

In the current study, adults’ implicit theories about social skills were manipulated and psychological and physiological responses to an actual SET (created in the lab) were investigated. The psychological responses included social self-esteem (a domain-specific component of self-esteem that focuses on one’s sense of *social* self-worth), spontaneous mentions of concerns about one’s social skills, and rumination. The physiological response assessed was heart rate variability (HRV). These specific variables were chosen because findings from prior studies have identified them as important and potentially harmful responses to SET, as detailed earlier (Smith et al., 1997; Dickerson and Kemeny, 2004; Gruenewald et al., 2004; Christian and Stoney, 2006; Dickerson et al., 2008; Zoccola et al., 2008; Bosch et al., 2009; Lehman et al., 2015). The primary goal of the current investigation was to determine whether implicit theories might shape the degree to which individuals engage in these potentially harmful responses to SET.

In general it was expected that relative to those induced to have entity theories, those induced to have incremental theories would exhibit a more adaptive set of responses to SET. Three main hypotheses were tested. First, it was hypothesized that, relative to those induced to have entity theories, those induced to have incremental theories would report fewer concerns about their social skills and less of a drop in social self-esteem in the face of SET. In situations of SET, they may be less likely to internalize the sense of social failure, thus protecting their sense of self-worth. For these individuals an instance of social failure would represent an opportunity for growth rather than a signal of stable and uncontrollable social skills deficits. Research on children suggests that incremental theorists are less likely to generate trait and ability attributions and blame themselves for failures (Erdley et al., 1997). Additionally, there is some evidence suggesting that incremental theories may protect one’s sense of self in the context of SET. Specifically, when individuals imagined or recalled rejection by a romantic partner (a type of SET), those with incremental theories were less likely to report incorporating the rejection into their self-*definition* (Howe and Dweck, 2016). Incremental theories might also be protective against the effects of SET on one’s sense of self-*worth*, specifically on one’s social self-esteem, as this domain of self-esteem is highly relevant to SET situations. In contrast, those induced to have entity theories should view negative evaluations by others as a relatively permanent state of affairs that is unlikely to improve, given their belief that social skills are fixed. For these individuals an instance of social failure is a reminder of stable and uncontrollable social skills deficits. Thus, relative to those with incremental theories, they should be more likely to internalize SET and suffer decrements in self-worth. For these same reasons they should also be more likely to report heightened concerns about their social skills in the face of SET.

Second, it was hypothesized that relative to those induced to have entity theories, those induced to have incremental theories would experience less rumination following SET. Although little work has been done to investigate the association between implicit theories and rumination, some findings suggest that those with incremental theories will dwell less on a difficult situation (Diener and Dweck, 1978). Additionally, entity theories of social skills may lead people to view situations of SET as uncontrollable, given that they do not believe they can improve their social skills in order to better manage these situations. Research suggests that when SET is perceived as uncontrollable it leads to increased rumination (Dickerson and Kemeny, 2004). Thus, relative to those with incremental theories, those with entity theories about social skills should be more likely to ruminate following SET.

In addition to the psychological outcomes discussed above it was predicted that, relative to those with entity theories, those with incremental theories would exhibit more adaptive physiological outcomes in response to SET. Specifically, those induced to have incremental theories would experience higher levels of HRV in response to SET. HRV refers to the variability present in the periods between consecutive heartbeats or the fluctuation in time intervals between consecutive heartbeats. Higher HRV is considered healthy because it suggests the cardiovascular system is responding to demands in an adaptive way rather than remaining stuck in a pattern (Thayer and Sternberg, 2006). Low HRV is associated with increased risk of cardiovascular disease (Curtis and O’Keefe, 2002; Yoo et al., 2011) and all-cause mortality (Thayer and Lane, 2007; Thayer et al., 2010). Thus, any association between implicit beliefs and HRV responses to SET are meaningful and may have implications for health.

Why might those with incremental theories be expected to have higher HRV in the face of SET? Those with incremental theories generally appraise challenging situations in a less threatening way and engage in more effective self-regulation. This may manifest itself in regulation of physiological responses to SET, specifically regulation of HRV. In fact, prior research suggests that those with incremental theories have a more adaptive cardiovascular response to SETs relative to controls (Yeager et al., 2016). The association between cardiovascular reactivity and entity theories has not been investigated but it is reasonable to believe that entity theorists would have difficulty regulating physiological responses to SET. The combination of SET and lack of control has been found to have synergistic effects on physiological reactivity (Dickerson et al., 2009). Given that those with entity theories about their social skills will experience SET as an instance of social failure that cannot be remedied with effort, they will essentially feel deprived of control. Relative to those with incremental theories, the experience of SET may have stronger effects on their physiological response.

## Method

### Participants

Participants were 124^1^ undergraduate students (26 males, 96 females, 2 rather not say) between the ages of 18 and 26 (*M* = 18.98, *SD* = 1.23). Participants were students at a university in Los Angeles, CA. Participants received either course credit or a $20 gift card for their participation.

### Procedure

Participants were recruited through the university subject pool and through signs posted on campus for a two-part study investigating responses to online social situations. They were told that they would engage in an online interaction and that physiological measurements would be assessed throughout. Prior to data collection, this experiment received approval from the Institutional Review Board at the university where data was collected (approval code: LMUIRB2016FA16).

### Online questionnaire session

Participants electronically signed a consent form and completed an online questionnaire that contained demographic measures. Participants were also informed in advance that electrocardiogram (ECG) recordings would be taken during the subsequent laboratory session.

### Experimental session

One to 2 weeks after completing the online questionnaire participants reported individually to a laboratory on the university campus for a one-hour experimental session. Upon arrival, they were met by a research assistant and told that during the experimental session they would read about psychological research on the social self, then engage in a short online interaction with other participants, and finally answer questions regarding their thoughts and feelings about the online interaction situation. They were led to believe that there were two other participants in adjacent rooms. In reality, there were no other participants and experimenters posed as other participants during the online interaction portion of the study.

Before engaging in any laboratory activities, the experimenter affixed three electrodes to the participant’s torso so that ECG recordings could be taken continuously to assess HRV. For the next 5 min participants sat quietly to allow baseline HRV measurements to be recorded. Next participants completed a questionnaire assessing social self-esteem (to serve as a measure of pre-interaction social self-esteem) and expectations about the how well the upcoming interaction would go. Because individual differences in expectations about the online interaction might affect participants’ responses this was used as a control variable. Participants were then exposed to a manipulation of implicit theories about social skills. They were either led to believe that social skills were more malleable or less malleable. Specifically, they were randomly assigned to read one of two online articles that were ostensibly from Psychology Today, and designed to induce either entity or incremental theories about social skills. This was based on a manipulation used by Howe and Dweck (2016) to manipulate implicit theories of *personality*. In the current study it was modified slightly to manipulate implicit theories about *social skills*. The entity version of this article described research supporting the idea that one’s social skills are established early on and are difficult to change once one enters adulthood. The incremental version described research supporting the idea that one’s social skills are malleable and can be improved throughout life. Both articles are included in the Appendix. After reading the article participants were presented with an attention check question to make sure they carefully read the article.

Next, participants were led to believe that there were two other participants in other lab rooms and that they would all take part in an online text-based chat interaction via Skype. They were told that the goal of the chat was to learn about what people find attractive or likeable in an online setting. They were told that each participant would be assigned to a specific role for the interaction. One participant would be randomly assigned to provide answers to questions (e.g., “What is one of your most important values?,” “What is the best part about your personality?”) and the two other participants would rate the participant on likeability following their response to each question. Finally, at the end of the interaction each rater would give an overall likeability rating for the participant who was answering the questions.

In fact, there were no other participants taking part in the study and the actual participant was always selected to answer the questions. The likeability ratings were actually provided by experimenters (posing as the two other participants in the chat) and they consistently gave low likeability ratings to the participant in order to create a situation of SET. Likeability ratings following the participant’s response to each question ranged from one to three stars (out of five possible stars, where five stars indicated very high likeability). All participants received identical low likeability ratings. At the end of the online interaction an overall likeability rating was given and the participant always received two stars from one rater and three stars from the other rater.

**Figure 1:**
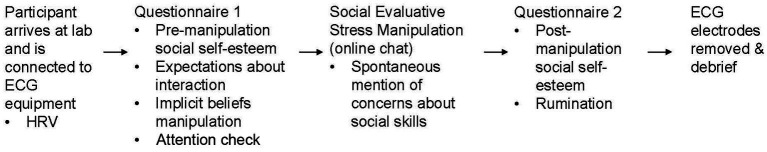
Overview of study timeline.

After the online interaction participants completed an online questionnaire assessing rumination and post-interaction social self-esteem. ECG electrodes were then removed and participants were carefully debriefed. A timeline of the experimental session is provided in Figure 1.

### Measures

#### Social self-esteem

Social self-esteem was assessed using the 7-item social self-esteem subscale from Heatherton and Polivy’s (1991) State Self-esteem Scale. This is a domain-specific measure of one’s self-esteem with regard to social skills and abilities (e.g., “Right now I am concerned about the impression I am making.,” “I am worried about what other people think of me.”). Social self-esteem was assessed at the beginning of the session (*M* = 4.02, *SD* = 0.82, α = 0.88) and following the online chat interaction (*M* = 3.57, *SD* = 1.07, α = 0.94) so that baseline levels of social self-esteem could be controlled for. Items were rated on a scale ranging from 1 (*not at all*) to 5 (*extremely*) and the mean for all items was computed for each participant.

#### Rumination

Rumination was assessed following the online chat (*M* = 3.47, *SD* = 1.42, α = 0.84) using a five-item scale assessing rumination. This scale was created by choosing individual items from two well-known rumination scales (e.g., Trapnell and Campbell, 1999; Treynor et al., 2003) to create statements that made sense in the context of the scenario created for the study (namely a stressful online chat). Participants were asked to indicate how frequently they engaged in certain cognitions *during and after the online social interaction* (e.g., “How frequently did you rehash part of the online interaction in your mind?,” “How frequently did you ruminate or dwell on part of the interaction?”). Participants responded using a scale that ranged from 1 (not at all) to 7 (often).

#### Concerns about social skills

Participants’ online chat transcripts were coded for spontaneous mentions of concerns about their social skills. Two raters, who were blind to participant condition, coded each transcript. Participants received a rating of zero if they made no reference to concerns about their social skills in the online chat and a rating of one if they mentioned concerns about their social skills. An example of a statement that illustrated concerns about social skills was “Sometimes I can be abrupt and it can come off as rude, but I find this to be because I want to get to the end result/answer faster. If I could change this I know it would make for less arguments or making people upset.” Interrater agreement was 90% and disagreements were settled by the author.

#### HRV

ECG signals were measured continuously using Biopac MP150 hardware with an ECG100C amplifier module (Biopac Systems, Inc., USA, using a high pass filter of 1.0 Hz, a low pass filter of 35 Hz, and a sampling rate of 1,000 Hz). Electrodes were affixed below the participant’s right and left clavicles as well as on the lower left abdomen over the ribcage. This placement was chosen to minimize artifacts due to movement when participants had to use one hand to type their answers to the questions posed in the online chat.

Heart rate waveforms were created in Acqknowledge 4.4 (Biopac Systems Inc., USA). They were visually inspected and edited to exclude artifacts (e.g., errors in the automatic detection of R-waves). Because the ECG signal is highly sensitive to movement, some physiological data could not be scored due to loss of signal or noisy signals, leaving 97 participants with intact HRV values for all time periods. After artifact removal, spectral power estimates were computed. Mean high frequency HRV (ms^2^) was computed for each of four time periods. High frequency heart rate variability (HF-HRV) was defined as power in the frequency range of 15 Hz to 0.4 Hz, in accordance with guidelines set forth by the Task force of the European Society of Cardiology and the North American Society of Pacing and Electrophysiology (1996). High frequency HRV was computed for each interval, as this frequency domain measure is preferred to time domain measures in the case of short-term recordings (Task force of the European Society of Cardiology and the North American Society of Pacing and Electrophysiology, 1996). Each of the four time periods lasted for approximately 5 min, with some minor individual variability. The first assessment of baseline/resting HF-HRV was calculated as the average of the final 5 min before the online chat. This represented resting HF-HFV once the participant had acclimated to the lab setting. The second assessment was calculated as the average of the first 5 min of the online chat. The third assessment was calculated as the average of the second 5 min of the online chat. The final assessment was calculated as the average of the 3 min following the online chat. A single outlying HF-HRV value (greater than three standard deviations from the overall mean) was winsorized (Tabachnick and Fidell, 2012) to diminish its effects. This value was replaced with the next highest value for the same participant.

#### Expectations about the upcoming interaction

Before the implicit theories manipulation, participants were asked about their expectations about the upcoming interaction (*M* = 4.7, *SD* = 1.07). This was measured with a single item that asked “How well do you think the online interaction with the other participants will go?” Items were rated on a 7-item scale ranging from 1 (not well at all) to 7 (extremely well). Responses to this question were entered in analyses to control for pre-existing expectations.

#### Attention check

Following the manipulation of implicit theories about social skills, participants were asked to read two short paragraphs and choose the one that best captured the main points of the article they had just read. One paragraph briefly described an entity perspective on social skills and the other one described an incremental perspective on social skills.

## Results

### Data analytic strategy overview

Analyses were conducted to investigate the association between condition (entity versus incremental) and each outcome variable. In all analyses condition was coded such that those in the entity theory condition received a code of zero and those in the incremental theory condition received a code of one. The variable assessing expectations about the upcoming interaction was entered as a control variable in all analyses except for the analysis of the attention check variable, as this variable should not have been affected by prior expectations, due to random assignment to condition and due to the relatively direct and objective nature of the question.

### Attention check

A chi-square analysis indicated that those who received the incremental theory manipulation were significantly more likely to choose the incremental theory article summary and those who received the entity theory manipulation were significantly more likely to choose the entity theory article summary *χ*^2^ (1, *n* = 147) = 70.84, *p* < 0.001. However, 23 participants answered the attention check question incorrectly (18 from the entity group and 4 from the incremental group). It was not surprising to find that it was harder to manipulate entity beliefs. Researchers have found that even when an entity perspective is manipulated participants still tend to skew toward the incremental perspective, given that this is the more common perspective (Rheinschmidt and Mendoza-Denton, 2014). Additionally, an incremental perspective is more socially desirable (Pomerantz and Kempner, 2013). Given how direct the attention check measure was, incorrect answers for the attention check suggested that a participant had rushed and neglected to read the implicit theories article carefully. An inspection of the duration of time spent reading the article by each participant supported this conclusion. These participants were dropped from analyses and all results reported below are based on the remaining 124 participants. This ensured that the final sample was composed of participants who had followed instructions and read the manipulation. The attention check was also clearly significant for this final group of participants *χ*^2^ (1, *n* = 124) = 124, *p* < 0.001, suggesting that they had carefully attended to the article they had been randomly assigned to read.

### Social self-esteem

Multiple linear regression analyses were conducted to investigate the association between condition and post-chat social self-esteem, controlling for pre-chat social self-esteem and expectations about the interaction. As shown in Table 1, there was no significant association between expectations about the interaction and post-chat social self-esteem and a significant association between baseline social self-esteem and post-chat social self-esteem. With regard to the variable of interest, condition was significantly associated with post-chat social self-esteem. The post-chat social self-esteem of those exposed to the incremental theory manipulation was significantly lower following SET (*M* = −0.34, *SD* = 0.733) than the post-chat social self-esteem of those exposed to the entity theory (*M* = −0.60, *SD* = 0.698).

**Table 1:** Linear regression analyses predicting each dependent variable from implicit theory condition.

Variable	Estimate	SE	*p*	*f^2^*
Post-chat social self-esteem				
Condition	0.124*	0.131	0.041	0.035
Expectations about interaction	−0.115†	0.061	0.064	
Pre-chat social self-esteem	0.765***	0.080	<0.001	
Rumination				
Condition	−0.182*	0.258	0.043	0.034
Expectations about interaction	0.106	0.118	0.239	

Values are standardized regression coefficients (β). Condition is dummy coded, where 0 = entity, and 1 = incremental.

† *p* < 0.10;

* *p* < 0.05;

*** *p* < 0.001.

### Rumination

Multiple linear regression analyses were conducted to investigate the association between condition and rumination, controlling for expectations about the interaction. As shown in Table 1, there was no association between expectations about the interaction and rumination. With regard to the variable of interest, condition was significantly associated with rumination indicating that those exposed to the incremental theory tended to ruminate less (*M* = 3.27, *SD* = 1.46) than those exposed to the entity theory (*M* = 3.78, *SD* = 1.30).

### Concerns about social skills

As shown in Table 2, logistic regression analyses revealed a significant association between expectations about the interaction and concerns about social skills. With regard to the variable of interest, analyses revealed a significant association between condition and the presence/absence of comments related to insecurity about one’s social skills during the online chat. 62% of those who had read the entity theory article spontaneously mentioned concerns about their social skills during the course of the chat. Only 41% of those who had read the incremental theory article mentioned concerns about social skills.

**Table 2:** Logistic regression analyses predicting concerns about social skills from implicit theory condition.

Variable	Estimate	SE	*p*	*Wald χ^2^*	*OR _entity_*
Concerns about social skills					
Condition	0.124*	0.131	0.041	0.035	1.535
Expectations about interaction	−0.858*	0.392	0.028	4.799	

Values are unstandardized regression coefficients (β). Condition is dummy coded, where 0 = entity, and 1 = incremental.

* *p* < 0.05.

### Heart rate variability

To investigate the linear association between implicit theory condition and HRV values growth curve modeling was conducted using Hierarchical Linear Model (HLM) software version 6.07 (Raudenbush et al., 2004). Significance tests were based on robust standard errors. The model below was the result of a process whereby successive model testing was conducted and the most parsimonious model was used. In the level 2 portion of the model condition is only used to predict slope (not intercept), since there was no reason to expect that condition would predict the intercept. To be thorough an alternative model was tested where condition was included as a predictor of intercept and, as expected, it did not predict the intercept. Thus, it was reasonable to assume that there were no differences in initial HRV levels between those in the entity and incremental conditions. Time was uncentered so that the intercept could be interpreted as the value of HRV at baseline. Using the coding scheme appropriate for testing for linear growth the four time points were coded as 0, 1, 2, and 3. The level 1 and level 2 models were specified as follows:

**Level 1 Model**


$${HRV}_{ti}=\pi_{0i}+{\pi_{1i}}^{*}\left(\mathrm{time}\right)+e_{ti}$$

**Level 2 Model**


$$\pi_{0i}=\beta_{00}+r_{0i}\text{.}$$

where HRV_ti_ represents the HRV value for individual *i* at timepoint *t* (where *t* is scaled from 0 to 3), π_0i_ represents the predicted intercept (or stated another way, the predicted initial HRV value at baseline or timepoint zero) for individual *i*, and π_1i_ represents the rate of change in HRV across timepoints for individual *i*. For the level 2 model, β_00_ represents the fixed intercept, β_10_ represents the fixed slope, β_11_ represents the change in slope as a function of condition, β_12_ represents the change in slope as a function of expectations about the interaction, and *r*_0i_ and *r*_1i_ represent residuals or random effects.

The results of the growth curve analyses (see Table 3) revealed a non-significant tendency toward a cross-level interaction of time and condition on the HRV slope (β_11_ = 81.92, *p* = 0.076). On average HF-HRV decreased for those in the entity condition and increased for those in the incremental condition. However, because this effect fell short of significance, a relationship between implicit theories and HF-HRV is not supported based on this data alone.

**Table 3:** Growth curve coefficients for the association between implicit theory condition and HRV values.

Fixed Effects	Coefficient	SE
Intercept, β_00_	901.48^***^	84.20
Slope, β_10_	−25.41	39.85
Condition, β_11_	81.92^†^	46.65
Expectations, β_12_	−5.90	20.31
Random Effects	Estimate	
*e*_ti_	502.97	
*r*_0i_	708.48^***^	
*R*_1i_	150.75^**^	

† *p* < 0.10;

*** *p* < 0.001;

** *p* < 0.01.

## Discussion

The overarching goal of this investigation was to gain a better understanding of the role that implicit theories play in shaping responses to SET. The findings suggest differences in responses to SET between those induced to have incremental theories about social skills and those induced to have entity theories about social skills. Relative to those induced to have entity theories, those induced to have incremental theories had higher levels of social self-esteem, fewer concerns about their social skills, and less rumination. Additionally, they exhibited a tendency toward healthier regulation of physiological responses, in terms of higher HRV. These findings are consistent with the findings seen in prior studies looking at the role of implicit theories in shaping responses to social situations for adults (Knee, 1998; Howe and Dweck, 2016). These findings suggest that relative to entity theories, incremental theories lead to a less harmful pattern of responses.

These findings have several theoretical implications. First, these findings allow for a better understanding of the role of implicit theories in shaping responses to SET, by manipulating rather than measuring implicit theories. Additionally, in most experimental studies on implicit theories only incremental theories are manipulated. In the current study both incremental and entity theories were manipulated, allowing for an investigation of the pattern of responses of both incremental theorists as well as entity theorists. Also, because implicit theories are domain-specific, the current study contributes to work aimed at understanding how implicit theories may affect responses in a social domain. Finally, the current findings contribute to research on strength in the face of SET. Most work focuses on deficits that lead people to be more vulnerable to SETs. The current study focuses not just on deficits that might leave one more vulnerable to SETs (i.e., entity theories) but also strengths that might buffer one from SETs (i.e., incremental theories). Socially threatening situations, such as SETs tend to be somewhat ambiguous and require some interpretation. The current findings suggest that when an individual’s interpretation of an SET is filtered through an entity theory this leads to negative outcomes. In contrast, an incremental theory leads to more positive outcomes, relative to those experienced by entity theorists. Thus, findings from the current study suggest that training individuals to have incremental versus entity theories, may lead to more adaptive responses to SETs.

These findings provide preliminary evidence that implicit theories may have implications for mental and physical health following SET. Relative to entity theories, incremental theories buffered individuals from the impact of SET by protecting their sense of self-worth, limiting their concerns or worries about their social skills, and leading to decreased rumination. Given the well-documented association between both high levels of rumination and low levels of self-esteem with negative physical and psychological outcomes (Orth et al., 2008, 2009; Guevara and Murdock, 2020) it is likely that those with incremental theories, who respond to SET with less rumination and less of a reduction in self-esteem may be partly protected from the harmful effects of SET. Additionally, given that rumination prolongs activation of the physiological stress response following SET (Zoccola et al., 2008; Zoccola and Dickerson, 2015), this potentially leaves incremental theorists at lower risk for decrements in physical health (McEwen, 1998; Sapolsky, 2007). Although the current study did not support a link between implicit theories and physiological responses to SET, the findings also do not definitively rule out such a relationship. This is discussed in more detail below.

## Limitations and future directions

Some limitations must be noted. First, future work should employ measures that provide more direct evidence that activation of implicit theories led to changes in the dependent variables assessed here. The manipulation used in the current study was closely modeled on an effective manipulation used by Howe and Dweck (2016). However, in the current study a more subtle attention check was used, instead of a more explicit manipulation check. This was done in order to avoid undoing the effects of the manipulation with an obtrusive measure (see Hauser et al., 2018 for a review of concerns about unintended effects of manipulation checks). Given the consistent pattern of group differences across several dependent variables this suggests that the manipulation was effective. However, future researchers may want to consider using a more explicit manipulation check to further confirm the causal nature of the effects presented here.

Second, the manipulation used in the current study was designed to allow for an investigation of the pattern of responses exhibited by entity versus incremental theorists. This manipulation was similar to that used by others researchers in this area and is based on the typical conceptualization of implicit theories as a bipolar construct where individuals either operate according to an entity or incremental theory. However, it should be noted that some researchers support investigating the possibility that entity and incremental theories are unipolar (Lüftenegger and Chen, 2017). If future work provides sufficient evidence suggesting that implicit theories are unipolar then, it would be important for researchers to begin investigating an additional control group of individuals who are not exposed to a manipulation of implicit theories, in addition to manipulating entity and incremental theories.

Additionally, clear conclusions cannot be made about the impact of implicit theories on HRV. The findings for the HRV analyses indicated a non-significant trend. This was likely in part due to the fact that some of the physiological data could not be used due to movement artifacts. This is a normal cost of physiological measurement in the laboratory. The net result of this data loss was lower power to detect a significant effect for the HRV analyses. Based on the data presented we must conclude that implicit theories do not affect HRV in response to SET. However, more work must be done in order to make more definitive conclusions about the effects of implicit beliefs on HRV following SET. Future studies should include other physiological responses in addition to HRV.

Finally, in the real world, it is likely that the relationship between implicit theories and the variables assessed in the current study is not unidirectional. And in fact, over time implicit theories may be shaped by factors such as one’s social self-esteem. Future work should employ a longitudinal design to further investigate the possible bidirectional nature of the relationship between implicit theories and the variables presented here.

Future research should also investigate long-term effects of implicit theories on responses to SET. The focus of the current study was on the role of implicit theories in shaping immediate responses to SET. Implicit beliefs may shape responses to SET over time in ways that were not captured here. For example, because those with incremental mindsets view SET as an opportunity to improve rather than as a threat, this may lead them to persist more in social situations and grow their social skills as a result. In fact, this pattern of responses is exhibited by incremental theorists in other domains, such as academic domains (for a review see Dweck, 2012). Future work should investigate whether incremental theorists optimize SET over time and therefore experience growth as a result of SET. Researchers should also investigate whether entity theories lead to avoidance of social situations over time. It has been suggested that decreased state self-esteem may act to reduce one’s interpersonal aspirations (Kirkpatrick and Ellis, 2004). Thus, for those with entity theories the cumulative effect of reductions in social self-esteem following SET situations may ultimately lead them to retreat from interactions that pose a risk of social threat.

## Conclusion

Past research has primarily focused on factors that leave individuals more vulnerable to SET and little work has been done to discover protective factors. Findings from the current study suggest that relative to entity theories about social skills, incremental theories about social skills may serve as a protective factor in the face of SET. As such, this study contributes to a small body of work that extends implicit theory research into the social domain. By gaining a better understanding of how implicit theories can shape responses to SET we can better understand how to promote adaptive responding in the face of social threat.

## Data availability statement

The datasets presented in this study can be found in online repositories. The names of the repository/repositories and accession number(s) can be found at: https://osf.io/r5ewp/?view_only=ca79ecb8b3f147a7b8607a425323361a.

## Ethics statement

The studies involving human participants were reviewed and approved by Loyola Marymount University Institutional Review Board. The patients/participants provided their written informed consent to participate in this study.

## Author contributions

The author confirms being the sole contributor of this work and has approved it for publication.

## Funding

This work was supported by the Bellarmine College of Liberal Arts and the Department of Psychological Science at Loyola Marymount University.

## Conflict of interest

The author declares that the research was conducted in the absence of any commercial or financial relationships that could be construed as a potential conflict of interest.

## Publisher’s note

All claims expressed in this article are solely those of the authors and do not necessarily represent those of their affiliated organizations, or those of the publisher, the editors and the reviewers. Any product that may be evaluated in this article, or claim that may be made by its manufacturer, is not guaranteed or endorsed by the publisher.

## Footnotes

^1^Based on prior studies a medium effect size (*f* = 0.25) was expected. An apriori power analysis was conducted using G*Power (Faul et al., 2007) and it was determined that the number of participants needed to achieve at least 80% power with a 5% false-positive rate was 128. The final sample in the current study before participants were dropped exceeded this number (*N* = 147) and the final sample after participants were dropped (*N* = 124) ended up being very close to this number.
