# Supplementary material for: Implicit theories shape responses to social-evaluative threat
Source: Front Psychol. 2023 Apr 27;14:1105721. doi: 10.3389/fpsyg.2023.1105721 (PMC10172678; doi:10.3389/fpsyg.2023.1105721)
Supplement: Supplementary file 1 [file Presentation_1.pdf]

Appendix

Text from Implicit Theories Articles

Text from Entity Theory Article

What is the key to happiness and achievement? Recent research reveals that our early social experiences influence our success in life. In this short article, we explore four critical factors that determine our personal qualities and social abilities early in life, and have long-lasting consequences.

The First Critical Factor to Success: Social Skills and Attachment

Classic research in developmental psychology shows that, at a young age, we develop specific patterns of interacting with others, called attachment styles. When an infant's relationship with his/her parents is troubled he/she forms an insecure attachment style. An infant who has a healthy relationship with his/her parents forms a secure attachment style. Infants who are insecure see the world as a more threatening place and tend to have less well-developed social skills, even years down the road. Without these abilities, insecure infants grow up into more anxious and less well-adjusted adults as certain behaviors and habits become more ingrained. Amazingly, research has shown that the socioemotional advantages that secure infants have translate into better relationships in adulthood, both romantic relationships and friendships.

The Second Critical Factor to Success: Developing Optimism

The information about secure and insecure attachment illustrates how our past experiences in social interactions can influence the way we act in the world today. Beyond attachment styles there are other basic orientations toward the world that work similarly.

For instance, you may have wondered whether the distinction between pessimists and optimists is meaningful, and whether someone who sees the glass as "half full" is actually different from someone who sees it as "half empty". In fact, recent research demonstrates that whether someone thinks like an optimist or a pessimist may directly affect his/her life. As early as elementary school, certain people tend to focus more on the negative. This pattern tends to persist throughout adulthood. These are the kinds of people who say, "If something bad can happen to me, it probably will" and expect the worst. Research shows that the earlier a person's optimism emerges the more social success they have in adulthood. These "early optimists" tend to have denser social networks and better close relationships.

The Third Critical Factor to Success: Developing Likeability

Have you ever met someone who just seemed "magnetic"? Recent research investigates people with a high "Likeability Quotient" or LQ, who seem naturally likeable. Most people who

seem naturally likeable report that they developed these social skills very early in life. As one interviewee said “I just always felt this drive to interact with other people, and early on I easily learned how to connect with people from all walks of life.” Likeability seems to be a characteristic that is developed in young adulthood, as people with high LQ seem to “just easily understand others”. People with high LQ are more likely to be leaders and to succeed at work, for example, getting higher ratings in interviews and more job promotions than people with a low LQ. This isn’t surprising, as people with a high LQ can effectively build relationships and establish trust with others.

The Fourth Critical Factor to Success: Developing a positive view of social stress

Many people think that social stress (stress that occurs in the context of social interactions with others) is bad and that it has negative effects. In fact, research suggests that social stress has some beneficial effects. For example, social stress motivates us to take action to fix the stressful situation and encourages problem-solving. It puts the brain in an optimal position to perform well in interactions with others. In fact, social stress has been found to lead to stress-related growth, whereby stressful interactions with others lead to increased mental toughness, increased awareness, improved social skills, and an increased sense of meaningfulness. A person’s view on social stress as either harmful or beneficial seems to be formed very early in life, perhaps even as young as the age of 3. People who have a positive view of social stress (ie., those who believe that social stress has some beneficial effects) are more likely to report smoother interpersonal interactions and better relationship outcomes.

In Conclusion

Overall, research on these four critical factors illustrates how important early experience is in shaping people’s lives and setting people up for interpersonal success. Basic distinctions, such as whether we choose to be securely or insecurely attached, optimistic or pessimistic, understand others or not, and view social stress as positive or negative can influence our friendships, close relationships, work, and academics. These are just a few important examples of how early experiences shape the way our lives play out.

Text from Incremental Theory Article

What is the key to happiness and achievement? Recent research reveals some key changes we can make to build our social success and improve our social lives at any point. In this short article, we explore four key ways in which we can develop our interpersonal qualities and social abilities to have long-lasting positive consequences.

The First Key Way to Success: Developing Social Skills

Classic research in developmental psychology shows that, at a young age, we develop specific patterns of interacting with others, called attachment styles. Some infants develop a secure attachment style, which is considered to be the healthiest attachment style. Other infants develop a less healthy attachment style, referred to as an insecure attachment style. The good news is that insecure infants do not necessarily grow up into more anxious or less well-adjusted adults. By making an effort to develop social skills, by working hard to form close friendships, or attending classes or counseling in social development, insecure people can change their behaviors and habits.

These changes translate into better relationship success years down the road in adult relationships, both romantic relationships and friendships.

The Second Key Way to Success: Developing Optimism

The malleability of secure and insecure attachment illustrates that we can overcome past experiences to influence the way we act in the world today. Beyond attachment styles, there are other basic orientations toward the world that work similarly.

For instance, you may have wondered whether the distinction between pessimists and optimists is meaningful, and whether someone who sees the glass as "half full" is actually different from someone who sees it as "half empty". In fact, recent research demonstrates that whether someone thinks like an optimist or pessimist may dramatically affect his or her life. As early as elementary school, certain people tend to focus more on the negative. These are the kinds of people who learn to say, "If something bad can happen to me, it probably will" and expect the worst. This pattern can persist throughout adulthood but research shows that at any age you can train your thinking to be more optimistic. People who "train optimism" tend to have denser social networks and better close relationships.

The Third Key Way to Success: Developing Likeability

Have you ever met someone who just seemed "magnetic"? Recent research investigates people with a high "Likeability Quotient" or LQ, who seem naturally likeable. Many people who seem naturally likeable report that they had to work hard to develop their social skills. As one interviewee said "I spent a long time figuring out how to interact with other people, and learned how to connect with people from all walks of life. I taught myself to view social interactions, even difficult ones, as opportunities for learning and growth". Likeability seems to be a characteristic that can be developed in young adulthood and beyond, as people build their LQ and hone their ability to understand others. People with a high LQ are more likely to be leaders and to succeed at work, for example, getting higher ratings in interviews and more job promotions than people with a low LQ. This isn't surprising, as people with a high LQ have learned effective ways to build relationships and establish trust with others.

The Fourth Key Way to Success: Developing a positive view of social stress

Many people think that social stress (stress that occurs in the context of social interactions with others) is bad and that it has negative effects. In fact, research suggests that social stress has some beneficial effects. For example, social stress motivates us to take action to fix the stressful situation and encourages problem-solving. It puts the brain in an optimal position to perform well in interactions with others. In fact, social stress has been found to lead to stress-related growth, whereby stressful interactions with others lead to increased mental toughness, increased awareness, improved social skills, and an increased sense of meaningfulness. People who recognize the beneficial effects of social stress are more likely to experience this growth. Although one's view on social stress as either harmful or beneficial begins to form early in life, recent research suggests that it is updated throughout life, even well into adulthood and that those who learn to develop a positive view of stress are more likely to report smoother interpersonal interactions and better relationship outcomes.

In Conclusion

Overall, we see that these four factors can shape people's lives and pave the way to interpersonal success. Basic distinctions, such as whether we choose to be securely or insecurely attached, optimistic or pessimistic, understand others or not, and view social stress as positive or negative can influence our friendships, close relationships, work, and academics. People who work hard to develop the skills discussed above learn to view interpersonal interactions as opportunities for growth and learning. These are just a few important examples of how we can shape the way our lives play out.
